# Commentary: Overcoming mTOR resistance mutations with a new-generation mTOR inhibitor

**DOI:** 10.3389/fphar.2016.00431

**Published:** 2016-11-22

**Authors:** Maurizio Renna

**Affiliations:** Department of Medical Genetics, Cambridge Institute for Medical Research, Wellcome Trust, Addenbrooke's Hospital, University of CambridgeCambridge, UK

**Keywords:** mTOR pathway, mTOR inhibitors, resistance mechanisms, cancer therapeutics, drug design

The mammalian target of rapamycin (mTOR) is a highly conserved serine-threonine kinase belonging to the phosphatidylinositol kinase-related protein kinases family, which plays a central role in regulation of cellular metabolism, growth, and proliferation (Figure 1A) (Wullschleger et al., 2006; Laplante and Sabatini, 2012). The PI3K-AKT-mTOR signaling axis is also one of the most commonly activated pathways in human cancers (Vivanco and Sawyers, 2002; Zoncu et al., 2011). A growing body of evidence identifies activation of mTOR signaling as a common occurrence in human cancers (Menon and Manning, 2008). Recently, activating mutations of mTOR itself have been identified through mining of human cancer genome databases (Hardt et al., 2011). Hyper-activation of mTOR signaling makes it an attractive target for therapeutic intervention and has driven the development of a number of mTOR inhibitors, many of which have progressed to clinical trials (Chiang and Abraham, 2007).

**Figure 1:**
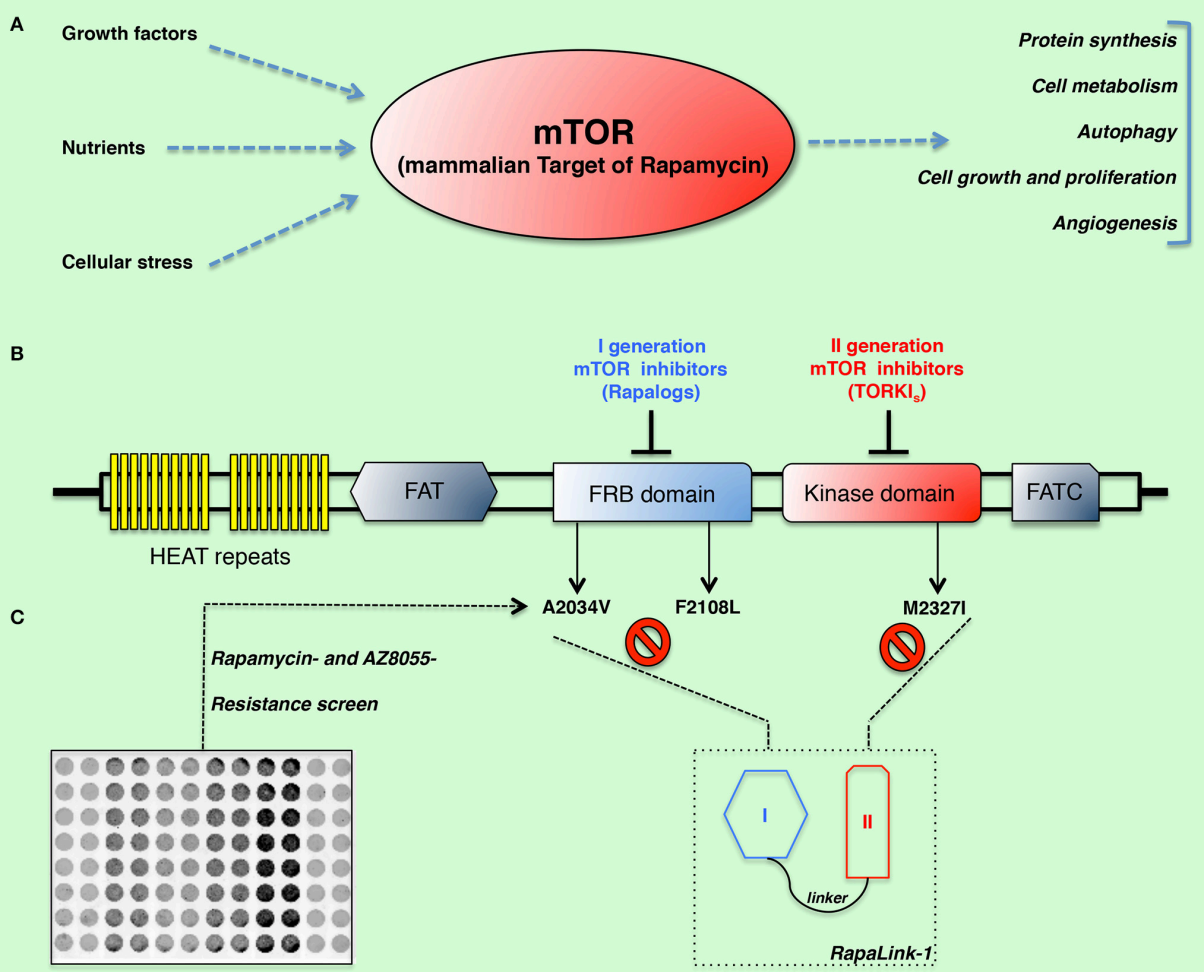
**(A)** Cells grow and proliferate when nutrients, growth factors, and the energy status trigger carbohydrate catabolism and the synthesis of essential building blocks such as proteins, nucleotides, and lipids. The mammalian target of rapamycin (mTOR) is a highly conserved serine-threonine kinase, which plays a central role in regulation of cellular metabolism, growth, and proliferation. The importance of mTOR in regulation of multiple cell functions is critical for development of cancer and its strong interaction with oncogenic pathways make this kinase an attractive target for therapeutic intervention. **(B–C)** Schematic representation of mTOR domains and mutations isolated in rapamycin- and AZD8055-resistant cells. By means of a resistance screen/deep sequencing combined approach, clinically relevant and I-II generation drug-resistant mTOR mutations (namely: A2034V, F2108L, and M2327I) were isolated. RapaLinks represent a novel class of bivalent mTOR inhibitors capable of overcoming resistance to existing first- and second-generation inhibitors (Rodrik-Outmezguine et al., 2016).

Among these agents, first-generation mTOR inhibitors such as rapamycin and rapalogs, inhibit mTOR by forming a complex with the immunophilin FKBP12, which then binds directly to mammalian TOR complex 1 (mTORC1), but not to mTORC2 (Guertin and Sabatini, 2009). Despite the mechanism by which FKBP12-rapamycin inhibits mTORC1 being not completely understood, the recently published structure of the mTOR catalytic domain provided additional insight, suggesting that the drug acts by blocking substrate recruitment (Yang et al., 2013). Limitations of rapalogs-based clinical strategies (and in particular, the strong immune-suppressive effects) have pushed toward the development of a second generation of mTOR inhibitors known as ATP-competitive mTOR kinase inhibitors (TORKIs), which target the kinase domain of mTOR and inhibit its catalytic activity. From a mechanistic point of view, the advantage of these drugs rely on the ability of inhibiting the kinase activity of both the TORC1 and TORC2 complexes, while also blocking the feedback activation of PI3K/Akt signaling (Thoreen et al., 2009). Numerous TKIs have been developed and several of them are currently being tested in clinical trials (Benjamin et al., 2011). Despite the many advantages of TORKIs, some drawbacks do exist. Although a number of cancers respond to mono-therapy treatment with rapalogs and TORKIs have been proved effective in rapamycin-insensitive cell lines, resistance remains a major concern (Feldman and Shokat, 2010). Another downside of TORKIs is their potential toxicity, which raises concerns about their long-term efficacy. Given the limitations of currently available inhibitors, new approaches toward the mTOR targeting are object of intense investigation. Combinatorial strategies may provide a way to overcome such resistance and therefore improve efficacy of mTOR targeting agents in the clinical context (Feldman and Shokat, 2010).

In a recent report, Kevan Shokat and collaborators describe the development of a third class of mTOR inhibitors that overcomes resistance to existing first- and second-generation inhibitors by exploiting the juxtaposition of two drug-binding pockets (hence, the eponymous RapaLink) (Rodrik-Outmezguine et al., 2016). By means of a resistance screen performed in MCF-7 cells they identified three somatic mutations within mTOR conferring resistance to either rapamycin (namely, A2034V and F2108L, both located in the FRB-FKBP12-rapamycin-binding- domain) or to the ATP competitive inhibitor AZD8055 (located in the kinase domain, at the M2327I position) (Figures 1B,C). The clinical relevance of these mutations is supported by a case report of a patient under treatment with everolimus who acquired the identical F2108L *MTOR* mutation after relapse (Wagle et al., 2014). More importantly, the hyperactive M2327I mutation (as well as other *MTOR* kinase domain mutations) has been identified in drug-naive patients (Grabiner et al., 2014). Such mutations might therefore impact the sensitivity to ATP-competitive mTOR inhibitors of some cancerous cells, regardless of the therapeutic regimen. In contrast to the FRB-domain mutations, which exhibit a similar mechanism of resistance by disrupting the interaction of mTOR with the FKBP12-rapamycin complex and ultimately the drug binding, the M2327I mutation confers hyperactivity to the kinase by an allosteric mechanism. These observations led the authors to develop a modeling approach aimed at overcoming drug-resistant mutations in either the FRB or the kinase domain. In principle, a bivalent mTOR inhibitor consisting of a rapamycin-FRB-binding element appropriately linked to a TORKi would be expected to inhibit the FRB-domain mutants because the TORKi-binding site would provide high-affinity recognition. Such an inhibitor would be similarly effective against the kinase domain mutations by virtue of an intact rapamycin-binding site, thus overcoming point mutations that diminish drug binding or that hyper-activate the kinase. In order to test such hypothesis, they generated bivalent molecules constituted by rapamycin and the highly selective TORKi MLN0128, separated by an inert chemical linker so that the resulting inhibitor could simultaneously bind to both sites. RapaLink_s_ exerted a strong signaling and growth inhibition both *in vitro and in vivo*, at levels comparable to rapamycin or a combination of rapamycin with MLN0128. Strikingly, the inhibitory effect of these drugs held true in both F2108L mTOR- and M2327I mTOR-expressing cells, as well as in the respective mouse xenograft models. Additionally, cells treated with RapaLink did not develop resistance to the drug for the 9-month period of the study, as opposed to cells treated with first- or second-generation mTOR inhibitors, which evolved resistance within 3 months.

To summarize, mTOR inhibition remains an attractive therapeutic option for the treatment of cancer and has the potential to play an increasingly prominent role in future treatment strategies. Although additional studies are required to determine their specific role in the clinical setting, mTOR inhibitors represent a promising therapeutic option for the treatment of cancer. Indeed, encouraging data from preclinical studies resulted in the initiation of multiple clinical trials. It is conceivable to speculate that this new class of mTOR inhibitors might be beneficial in patients bearing naïve hyperactive *MTOR* kinase domain mutations, who originally respond to rapalogs, as well as in those that have acquired resistance to rapalogs or ATP-competitive inhibitors, or both. To evaluate its potential as a cancer therapy, clinical trials will have to be performed. In particular, it will be of critical importance to assess whether these compounds cause the insurgence of novel resistance mutations, as well as to evaluate whether a higher degree of mTOR inhibition impact on their toxicity profile. Finally, it is important to note how the design of bivalent inhibitors for therapeutic purposes has not been exploited in protein kinase inhibitor design, so far. Hence, the methodological approach employed in this study could pave the way toward the design of novel bivalent inhibitors and be applied, more generally, to other disease-relevant signaling pathways.

## Author contributions

MR conceived the work, prepared the figure, wrote and edited the manuscript.

### Conflict of interest statement

The author declares that the research was conducted in the absence of any commercial or financial relationships that could be construed as a potential conflict of interest.
